# Access and Barriers to Healthcare Vary among Three Neighboring Communities in Northern Honduras

**DOI:** 10.1155/2012/298472

**Published:** 2012-06-19

**Authors:** Catherine A. Pearson, Michael P. Stevens, Kakotan Sanogo, Gonzalo M. L. Bearman

**Affiliations:** School of Medicine, Virginia Commonwealth University, Richmond, VA 23298, USA

## Abstract

*Objective*. The aim of this study is to describe and compare access and barriers to health services in three proximal yet topographically distinct communities in northern Honduras served by the nonprofit organization the Honduras Outreach Medical Brigada Relief Effort (HOMBRE). *Methods*. Study personnel employed a 25-item questionnaire in Spanish at the point of care during HOMBRE clinics in Coyoles, Lomitas, and La Hicaca (*N* = 220). We describe and compare the responses between sites, using Chi-squared and Fisher Exact tests. *Results*. Respondents in Lomitas demonstrated the greatest limitations in access and greatest barriers to care of all sites. Major limitations in access included “never” being able to obtain a blood test, obtain radiology services, and see a specialist. Major barriers were cost, distance, facility overcrowding, transportation, being too ill to go, inability to take time off work, and lack of alternate childcare. *Conclusions*. Despite being under the same local health authority, geographically remote Honduran communities experience greater burdens in healthcare access and barriers than neighboring communities of the same region.

## 1. Background

Short-term medical missions are an increasingly popular method for delivery of healthcare to developing nations, including Honduras [1–4]. The medical literature sparsely details best practices and optimization of clinical services for short-term mission trips. A number of studies have identified the importance of needs assessments and provider knowledge of local infrastructure for the success of sustainable, mission-based healthcare [1, 4, 5]. 

It is documented that Honduras lacks adequate access to health services: 83% of Honduras is without health insurance and 30% is without health care as of 2007 [6]. Infant mortality, a standard measure of population health, is 25 per 1,000, 10 more than the regional average [7]. The reasons for healthcare limitations in Honduras are multiple, including inadequate numbers of physicians (0.8 per 1,000 persons), variable medication supplies, gaps in infrastructure, financial constraints, and sociopolitical factors [8, 9]. 

Rural Honduras, home to 51% of the population, is particularly limited in both access to basic health infrastructure such as improved drinking water and sanitation, as well as access to healthcare [6, 7], with services limited to small health posts staffed by auxiliary nurses [6, 8]. 

Leon reported healthcare users in Honduras feeling they are “passive recipients of services.” They must “walk long distances to catch a bus or get a ride” only to find the clinics are full: “we are told to come back another day.” Rural Honduran populations in particular consider themselves disenfranchised, and “are preoccupied almost exclusively with obtaining a basic modicum of health care and medication in their communities” [10]. Though it is documented that Honduras as a nation lacks adequate healthcare access, and there is disparity between rural and urban sites, little is known about variations within rural, proximal communities.

**Figure 1:**
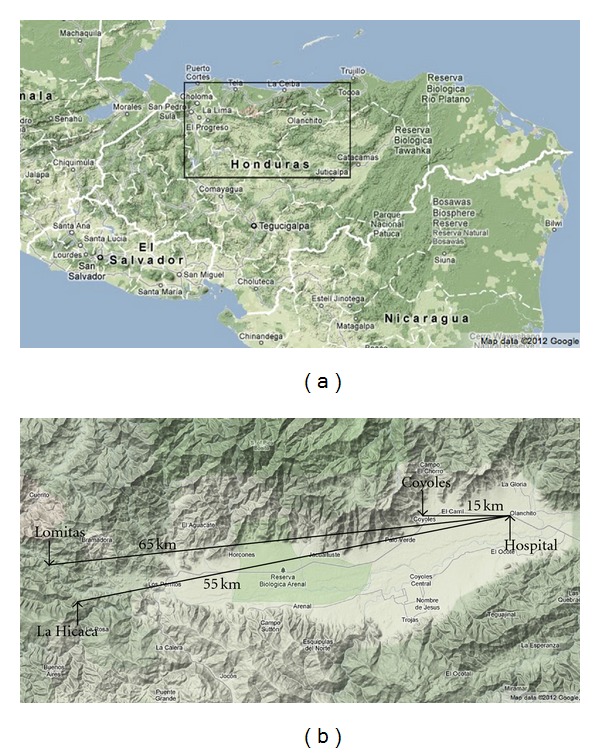
(a) Map of Northern Honduras (©  Google 2012) [11]. (b) HOMBRE Clinic Sites in the Department of Yoro, Honduras (©  Google 2012) [12].

The nonprofit organization, Honduras Outreach Medical Brigada Relief Effort (HOMBRE), has operated health clinics in three proximal yet geographically distinct communities in the northern district of Yoro, Honduras for five years. One site (Coyoles) lies in a flat, suburban landscape 15 kilometers from the nearest hospital. La Hicaca and Lomitas are mountainous and rural, 55 and 65 kilometers, respectively, from the nearest hospital (Figure 1). While Coyoles has a public bus system and paved roads, La Hicaca and Lomitas experience more variable terrain and limited infrastructure. Lomitas is the most remote with the greatest infrastructure limitation. The clinics in La Hicaca and Lomitas attract patients from a number of surrounding villages and homes. All communities are under the auspices of the same local health ministry.

Needs assessment studies administered in 2008 and 2009 [13], as well as the Adult Health Initiative database [14, 15] in Coyoles and La Hicaca, demonstrated differences in health status and burden of both chronic and infectious diseases between the communities of La Hicaca and Coyoles. Whitney et al. reported a decreased odds ratio for obesity, diagnosis of gastroesophageal reflux disease, and receipt of analgesics, multivitamins, and proton pump inhibitors/H2 blockers in the population of La Hicaca, as compared to Coyoles [16]. Despite geographic proximity and oversight by the same regional Ministry of Health, differences in disease prevalence and health pressures within these communities may be associated with differences in access to health services.

## 2. Objectives

The purpose of the study was to describe and compare barriers to healthcare access in the proximal yet geographically distinct northern Honduran communities of Lomitas, La Hicaca, and Coyoles. The study examined the potential influence of topography on healthcare access within neighboring communities under the same Ministry of Health with the goal of better focusing limited medical services during future relief trips. 

## 3. Methods

A 25-item questionnaire was approved by the Institutional Review Board of Virginia Commonwealth University and was administered at HOMBRE mobile clinic sites in Coyoles, Lomitas, and La Hicaca in June 2011 (see Supplementary Material available online at doi: 10.1155/2012/298472). Three items queried demographic information including sex, age, and home village. The 22 remaining questions were divided into three groups. Seven multiple-choice questions addressed utilization and access to healthcare services and medication. Six questions explored access to diagnostic testing and specialty medical services. Responses included a Likert scale of “never”, “sometimes”, “almost always”, and “always”. Nine questions evaluated the presence of common barriers to accessing healthcare. A Likert scale was used to identify a given barrier as presenting “no problem”, “some problem”, or a “big problem” for the respondent. Barriers to care included cost, distance to a health clinic, availability of transportation, healthcare facility overcrowding, lack of trust in one's healthcare provider, feeling too ill to seek care, inability to take time off work, no alternate childcare, and “other.” Each survey response was given a corresponding numeric code for data entry.

Eligible subjects included any Spanish speaking persons aged 18 years or above seeking care at the three June 2011 HOMBRE mobile field clinics. The study was conducted over eight clinic encounter days, each clinic day lasting eight hours. Five hours were spent daily conducting the interviews. Three study personnel conducted structured interviews using convenience-sampling methodology. The author (CP) was the primary interviewer at every site, with additional support by the two other trained, bilingual interviewers. All study personnel were trained and observed by the author (CP) before conducting interviews independently. Each survey was administered as a structured individual interview in Spanish at the point of care. Survey responses were collected on paper and subsequently entered into a secure mobile computer on site, using the data entry codes. 

Survey responses were analyzed using SAS statistical software (version 9.2, SAS Institute, Inc., Cary, NC). Descriptive analysis of the data was conducted using mean values, frequency counts, and percent response where applicable. A Chi-squared test of significance was employed to determine a difference in response across the three communities. Fisher Exact test was employed when applicable to small sample size. Pairwise comparisons were employed to evaluate each site to one another.

## 4. Results

### 4.1. Population Characteristics

Two hundred twenty surveys were completed. Sixty-four percent (*n* = 140) of respondents were from Coyoles, 23% (*n* = 50) were from Lomitas, and 14% (*n* = 30) were from La Hicaca. The mean age of survey respondents was 44 years. Of the respondents, 78% (*n* = 171) were female and 22% (*n* = 49) were male. Age and gender distributions displayed no statistically significant differences between the three sites. More than 30 home villages were reported. 

### 4.2. Healthcare Use and Access

Seventy percent (*n* = 35) of respondents in Lomitas reported no contact with a healthcare provider in the last 12 months versus 43% (*n* = 13) in La Hicaca and 28% (*n* = 39) in Coyoles (*P* < 0.0001).

**Figure 2:**
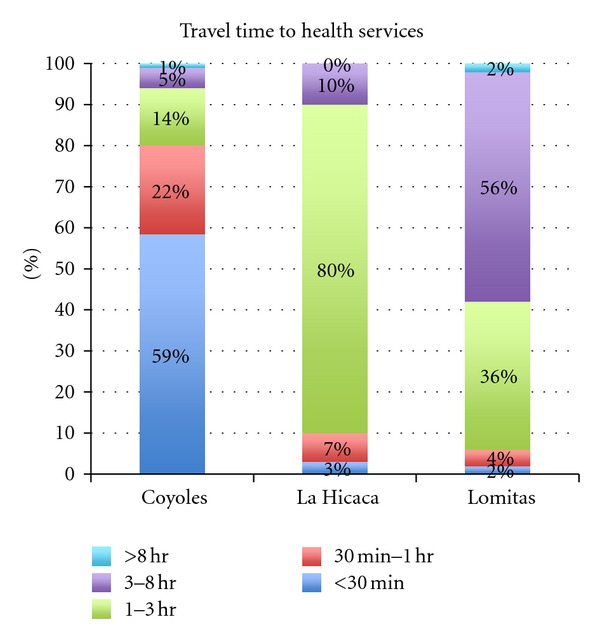
Travel time to health services. The most frequently reported trip lengths per site were <30 minutes in Coyoles (59%, *n* = 82), 1–3 hours in La Hicaca (80%, *n* = 24), and >3 hours in Lomitas (58%, *n* = 29).

Figure 2 summarizes response to healthcare use and access among the three communities. The majority (59%, *n* = 82) of respondents in Coyoles accessed their health provider in less than thirty minutes. The majority (80%, *n* = 24) of respondents in La Hicaca accessed their health provider in 1–3 hours, while the majority in Lomitas (58%, *n* = 29) reported >3 hours travel to access medical care.

The mode of transportation used to access medical services varied between the sites. The majority of respondents in Lomitas accessed healthcare by foot (70%, *n* = 35), while travel by automobiles was most common in La Hicaca (60%, *n* = 18) and use of public bus was most reported in Coyoles (51%, *n* = 71).

Thirty-eight percent (*n* = 84) of survey respondents cited one reason for seeking healthcare. The most common reasons cited for visiting a healthcare provider were “when ill” (95%, *n* = 208), “preventive care” (51%, *n* = 112) such as vaccines or cytology, “obtain medications” (43%, *n* = 94), “prenatal care” (17%, *n* = 37) and “other” (8%, *n* = 17). “Other” included screening tests such as blood pressure and blood glucose checks.

Table 1 summarizes access to blood testing, radiology, and specialists across the sites. Significant differences were observed between the sites for reported access to blood testing, radiography, and medical specialists, with Lomitas demonstrating the highest percent of respondents “never” having access to these medical services. Ability to obtain a blood test or see a specialist were not significantly different between La Hicaca and Coyoles.

### 4.3. Barriers to Accessing Health Services

Table 2 summarizes the barriers to healthcare access deemed “big problems” by respondents. Significant differences were observed between sites in the percentage of respondents reporting cost, distance, availability of transportation, being too ill to go, not being able to take time off work, or having no alternate childcare as “big problems”. 

There were no significant differences between La Hicaca and Coyoles in the percentage of respondents reporting cost or having no alternate childcare as “big problems”. Additionally, there was no significant difference between La Hicaca and Lomitas in the percent listing “too ill to go” as a big problem. 

The majority of respondents at all three sites reported facility crowding as a “big problem”. 

Neither mistrust in one's healthcare provider nor poor comprehension of medical recommendations was identified as a significant barrier to healthcare. Eighty percent (*n* = 174) of respondents study-wide reported “no problem” in trusting their healthcare provider. Likewise, 82% (*n* = 176) of all survey respondents reported “always” or “almost always” understanding physician recommendations. There was no statistically significant difference between sites regarding their trust of their healthcare providers.

## 5. Discussion

Short-term medical missions to Central America are popular. However, there is a paucity of literature evaluating healthcare access and detailing the role of medical missions in Honduras. The short-term medical mission HOMBRE has been serving three communities in northern Honduras for five years. Needs assessments conducted in 2008 and 2009 described clinical needs of the populations [13]. 

In this study, we describe and compare access and barriers to healthcare in Lomitas, La Hicaca, and Coyoles. All sites demonstrated substantial gaps in access to care. Individuals are often hours from a health provider, with limited access to transportation, diagnostic testing, and specialty services.

The widespread limitations to healthcare accessibility in Honduras have been previously reported [8, 14]. The Pan-American Health Organization has reported that 30% of Honduras is without healthcare access [8]. Leon et al. reported that the main concerns of healthcare users in Central America were “prompt access to trusted physicians, effective and inexpensive medication, and quality attention in public hospitals” [10]. Our study supports these previous findings. Forty percent of respondents in our study reported no contact with a health provider in the last year. Likewise, as in the Leon et al. study, the cost of care, time needed to access care, and healthcare facility overcrowding were all cited as major barriers to health services. 

Our study expands on the concept of healthcare variation between rural and urban settings in Central America and illustrates further disparities in healthcare access within closely neighboring, rural areas. Reasons for accessing health services were consistent throughout the study sites. Local health services were primarily used when ill only (95% of respondents), while only 51% of respondents reported using healthcare resources for preventive care. These findings suggest that preventive health services may be a potential focus for future short-term medical relief missions.

Significant differences in healthcare access were observed between all sites. Lomitas, the most rural and mountainous of the three locations, consistently reported the highest burdens in barriers to care. Cost, distance, transportation, facility overcrowding, ability to take time off work, and obtaining alternate childcare, were all reported more frequently as a “big problem” in Lomitas than in La Hicaca and Coyoles. Likewise, La Hicaca consistently demonstrated a higher percentage reporting a “big problem” for the distance, transportation, feeling too ill to seek care, and ability to take time off work than Coyoles. 

A similar trend was observed in reported ability to adhere to healthcare recommendations. More than 75% of Lomitas respondents reported a complete inability to access laboratory testing, radiology, or specialty care when recommended by a health provider. 

Coyoles, La Hicaca, and Lomitas are located 15, 55, and 65 kilometers from the nearest hospital, respectively, (Figure 1). While the study findings do correlate with the increasing distance to healthcare, the distance between La Hicaca and Lomitas is relatively small (approximately 10 kilometers). Thus, distance alone does not explain the significant differences reported in access and barriers to care in these communities. Other factors such as road infrastructure and topographical variation may contribute to the differences identified between these communities.

Limitations of our study include the use of multiple interviewers and convenience sampling methodology. Survey respondents may have been more motivated to both attend clinics and express their healthcare concerns, potentially biasing the results. Because the study was conducted among clinic attendees, the results excluded those unable to travel to the clinics for health or mobility reasons, and therefore may have underreported travel burdens. The majority (78%) of survey respondents were women. As a result, study participants may not be representative of their referent communities and of rural Honduran communities in general. Additionally, this study was conducted in a single region of Honduras, thus the data cannot be generalized to other Central American countries or communities. 

Our study has multiple strengths. The survey was developed in English, translated to Spanish and back translated to English to ensure internal validity during survey development. All surveys were conducted over the same relief trip and thus represented the communities at the same instant in time. Interviews were conducted on a personal basis, minimizing reader misinterpretation and overcoming the limitations in literacy in the community. Lastly, despite the use of three interviewers, all were trained under the same methodology and directly supervised during initial interviews.

Our study further supports the concept that rural, isolated communities face the greatest challenges in accessing healthcare. However, these data also demonstrate that nuanced barriers to care may differ significantly even between geographically proximal communities. More research is needed to evaluate novel mechanisms for increasing healthcare access to poor Central American communities. 

New technologies offer promising opportunities for overcoming healthcare access barriers in resource poor settings. Piette et al. provided evidence in 2010 that telemedicine and automated phone calls can have a positive effect on medication and appointment adherence in patients with chronic disease in low-income and low-access areas of Honduras [17]. The training of local community health workers to provide long-term primary care services is another promising area of focus [18]. Other efforts suggest that mobile pharmacies supplied by international nongovernmental organizations and run by local community members may function to provide medications to rural communities in Honduras [19]. However, studies have shown these to have many challenges of their own, including problems in sustainable management and quality of care [20, 21].

Our study supports the evaluation of topographical differences on health care utilization in rural communities. Geographic Information Systems (GIS) is a spatial mapping tool that is being increasingly used to map health disparities geographically [22, 23]. There is growing evidence of its utility in developing countries suggesting that GIS may be an effective tool for evaluating health disparities and barriers in rural communities [24–26].

Our study adds to the body of literature on Honduran and Central American medical relief work. Our findings suggest that topography may significantly impact medical access even in neighboring, remote communities. This finding underscores the need for medical relief trips to fully consider potential topographic influences on healthcare access even when rural communities are seemingly proximal and socioeconomically similar. Our findings highlight the importance of medical relief preparation and planning to best serve the needs of isolated, Honduran communities. Much of Honduras and Central America is populated in impoverished, rural, and mountainous areas. As such, nuances in access to healthcare, as a function of topography, call for targeted resource allocation and novel approaches to overcome barriers in healthcare delivery by short-term medical missions.

## Supplementary Material

Survey instrument, administered by individual interview at the point of care at the HOMBRE mobile clinic sites in June 2011.

## Figures and Tables

**Table 1:** Number of respondents reporting “never” being able to adhere to a given healthcare recommendation.

Number of respondents “never” able to adhere to a healthcare recommendation, % (*N*)			
	Lomitas	La Hicaca	*P* value

Obtain blood test	78% (39/50)	28% (8/29)	<0.0001
Undergo radiography	86% (43/50)	52% (15/29)	0.0009
See specialist	82% (41/50)	34% (10/29)	<0.0001

	Coyoles	Lomitas	*P* value

Obtain blood test	20% (27/138)	78% (39/50)	<0.0001
Undergo radiography	29% (40/136)	86% (43/50)	<0.0001
See specialist	37% (50/135)	82% (41/50)	<0.0001

	La Hicaca	Coyoles	*P* value

Obtain blood test	28% (8/29)	20% (27/138)	0.3347
Undergo radiography	52% (15/29)	29% (40/136)	0.0207
See specialist	34% (10/29)	37% (50/135)	0.7956

**Table 2:** Pair-wise comparisons of barriers to healthcare access identified as a “big problem”.

Barriers to healthcare access identified as a “big problem” % (*N*)			
	Coyoles	Lomitas	*P* value

Cost	52% (72/138)	88% (44/50)	<0.0001
Distance	37% (51/138)	92% (46/50)	<0.0001
Availability of transport	37% (51/138)	90% (45/50)	<0.0001
Crowded facility	66% (91/138)	94% (47/50)	0.0001
Too ill to go	40% (55/138)	82% (41/50)	<0.0001
Cannot take time off work	36% (49/138)	88% (44/50)	<0.0001
No alternate childcare	22% (31/138)	62% (31/50)	<0.0001

	Lomitas	La Hicaca	*P* value

Cost	88% (44/50)	63% (19/30)	0.0090
Distance	92% (46/50)	67% (20/30)	0.0039
Availability of transport	90% (45/50)	67% (20/30)	0.0096
Crowded facility	94% (47/50)	73% (22/30)	0.0094
Too ill to go	82% (41/50)	80% (24/30)	0.8244
Cannot take time off work	88% (44/50)	60% (18/30)	0.0037
No alternate childcare	62% (31/50)	34% (10/29)	0.0183

	La Hicaca	Coyoles	*P* value

Cost	63% (19/30)	52% (72/138)	0.2662
Distance	67% (20/30)	37% (51/138)	0.0028
Availability of transport	67% (20/30)	37% (51/138)	0.0028
Crowded facility	73% (22/30)	66% (91/138)	0.4343
Too ill to go	80% (24/30)	40% (55/138)	<0.0001
Cannot take time off work	60% (18/30)	36% (49/138)	0.0130
No alternate childcare	34% (10/29)	22% (31/138)	0.1716

## References

[1] Maki J, Qualls M, White B, Kleefield S, Crone R (2008). Health impact assessment and short-term medical missions: a methods study to evaluate quality of care. *BMC Health Services Research*.

[2] Kanter SL (2008). Global health is more important in a smaller world. *Academic Medicine*.

[3] Drain PK, Holmes KK, Skeff KM, Hall TL, Gardner P (2009). Global health training and international clinical rotations during residency: current status, needs, and opportunities. *Academic Medicine*.

[4] Suchdev P, Ahrens K, Click E, Macklin L, Evangelista D, Graham E (2007). A Model for sustainable short-term international medical trips. *Ambulatory Pediatrics*.

[5] Decamp M (2007). Scrutinizing global short-term medical outreach. *Hastings Center Report*.

[6] Pan-American Health Organization (2007). Country profiles: Honduras. *Health in the Americas*.

[7] World Health Organization (2010). *World Health Statistics*.

[8] (2004). *Exclusion in Health in Latin America and the Caribbean*.

[9] Smith HM, DeKaminsky RG, Niwas S, Soto RJ, Jolly PE (2001). Prevalence and intensity of infections of ascaris lumbricoides and trichuris trichiura and associated socio-demographic variables in four rural honduran communities. *Memórias do Instituto Oswaldo Cruz*.

[10] Leon M (2003). Perceptions of health care quality in Central America. *International Journal for Quality in Health Care*.

[11] Honduras, terrain map. http://www.google.com/maps.

[12] Department of Yoro Honduras, terrain map. http://www.google.com/maps.

[13] Stevens MP, Stevens LF, Elam K Medical needs assessment and infectious diseases concerns in rural Honduras-implications for medical relief planning.

[14] Hemrajani RH, Elam K, Morehouse B Top health concerns in rural Honduras following the introduction of clay water filters.

[15] Stevens MP (2010). *Optimizing Public Health Efforts on a Medical Relief Brigade to Northern Honduras: The Adult Health Initiative*.

[16] Whitney R, Stevens M, Bearman G Medical relief services in rural Honduras: an assessment of healthcare needs and delivery with a comparison of two neighboring communities. *Webmed Central Public Health*.

[17] Piette JD, Mendoza-Avelares MO, Milton EC, Lange I, Fajardo R (2010). Access to mobile communication technology and willingness to participate in automated telemedicine calls among chronically ill patients in Honduras. *The Official Journal of The American Telemedicine Association*.

[18] Rennert W, Koop E (2009). Primary health care for remote village communities in Honduras: a model for training and support of community health workers. *Family Medicine*.

[19] Fiedler JL, Wight JB (2000). Financing health care at the local level: the community drug funds of Honduras. *International Journal of Health Planning and Management*.

[20] Fiedler JL, Suazo J (2002). Ministry of Health user fees, equity and decentralization: lessons from Honduras. *Health Policy and Planning*.

[21] Chukwuani CM, Olugboji A, Ugbene E (2006). Improving access to essential drugs for rural communities in Nigeria: the Bamako initiative re-visited. *Pharmacy World and Science*.

[22] McLafferty SL (2003). GIS and health care. *Annual Review of Public Health*.

[23] Tanser F, Gijsbertsen B, Herbst K (2006). Modelling and understanding primary health care accessibility and utilization in rural South Africa: an exploration using a geographical information system. *Social Science and Medicine*.

[24] Tanser F, Hosegood V, Benzler J, Solarsh G (2001). New approaches to spatially analyse primary health care usage patterns in rural South Africa. *Tropical Medicine and International Health*.

[25] Noor AM, Gikandi PW, Hay SI, Muga RO, Snow RW (2004). Creating spatially defined databases for equitable health service planning in low-income countries: the example of Kenya. *Acta Tropica*.

[26] Fisher RP, Myers BA (2011). Free and simple GIS as appropriate for health mapping in a low resource setting: a case study in eastern Indonesia. *International Journal of Health Geographics*.

