# Supplementary material for: Access and Barriers to Healthcare Vary among Three Neighboring Communities in Northern Honduras
Source: Int J Family Med. 2012 Jun 19;2012:298472. doi: 10.1155/2012/298472 (PMC3388342; doi:10.1155/2012/298472)
Supplement: Supplementary file 1 — Survey instrument, administered by individual interview at the point of care at the HOMBRE mobile clinic sites in June 2011. [file 298472.f1.docx]

| Fecha: | Lugar: | Encuestador: |
| --- | --- | --- |

*Demografia*

1. Sexo: [ ] Masculino _1_ [ ] Femenino _2_
2. Edad: _________ años
3. Lugar de residencia:_______________

***Lugar habitual de cuidado medico***

1. ¿ Durante los últimos 12 meses, cuántas veces ha visitado o ha hablado con un profesional sobre su salud ?

| [ ] 0 _1_ | [ ] 1 _2_ | [ ] 2-3 _3_ | [ ] 4-9 _4_ | [ ] 10 o mas _5_ | [ ] No sé _9_ |
| --- | --- | --- | --- | --- | --- |

1. ¿ Qué lugar frecuenta para su cuidado medico?

| [ ] Médico local _1_ | [ ] Dispensario _2_ | [ ] Hospital _3_ | [ ] Misión médica _4_ |
| --- | --- | --- | --- |

1. ¿ Por qué medio se desplaza para hacer una consulta médica? (Elija todas las apropiadas respuestas)

| [ ] A pie _1_ | [ ] Por carro _2_ | | [ ] Motocicleta _3_ | | [ ] Transporte public _4_ | | [ ] Taxi _5_ |
| --- | --- | --- | --- | --- | --- | --- | --- |
| [ ] A caballo/ burro _6_ | | [ ] Bicicleta _7_ | | [ ] Otro medio _8_ | | [ ] No sé _9_ | |

1. ¿ Cuánto tiempo demora normalmente en llegar a un consultorio médico?

| [ ] <30 min. _1_ | [ ] 30 min – 1 hora _2_ | [ ] 1-3 hrs _3_ | [ ] 3-8 hrs _4_ | [ ] > 8 hrs _5_ |
| --- | --- | --- | --- | --- |

1. ¿ Si necesita buscar un medicamento, cuánto demora el viaje para buscarlo?

| [ ] En el mismo lugar donde recibo atención médica _9_ | | | | |
| --- | --- | --- | --- | --- |
| [ ] <30 min. _1_ | [ ] 30 min – 1 hora _2_ | [ ] 1-3 hrs _3_ | [ ] 3-8 hrs _4_ | [ ] > 8 hrs _5_ |

1. ¿ Por qué razones ve a un professional de la salud? (Elija todas las apropiadas respuestas)

| [ ] Cuando estoy enfermo _1_ | | [ ] Cuidado preventive (vacunas, citología,) _2_ | |
| --- | --- | --- | --- |
| [ ] Cuidado prenatal _3_ | [ ] Para recoger medicamentos _8_ | | [ ] Otro: _________________9_ |

1. ¿ Cuánto tiempo normalmente espera para ser atendido?

| [ ] <30 min. _1_ | [ ] 30 min – 1hora _2_ | [ ] 1-2 hrs _3_ | [ ] 2-5 hrs _4_ | [ ] > 5 hrs _5_ |
| --- | --- | --- | --- | --- |

***Identificando obstáculos para el cuidado médico***

A continuación se da una lista de posibles razones por las cuales no se puede acceder a prestaciones de salud Por favor indique cuales son las que le afectan a Usted:

|  | No es un problema _1_ | Un poco problematic _2_ | Un gran problema _3_ |
| --- | --- | --- | --- |
| 8. Costo de la prestación o del medicamento |  |  |  |
| 9. Distancia a la clínica |  |  |  |
| 10. Disponibilidad de transporte |  |  |  |
| 11. El lugar es demasiado atestado |  |  |  |
| 12. No tengo confianza en la persona que me atiende |  |  |  |
| 13. Demasiado enfermo para ir |  |  |  |
| 14.No puedo ausentarme del trabajo |  |  |  |
| 15. No tengo quien cuide los niños |  |  |  |
| 16. Otra razón: _________________ |  |  |  |

Por favor indique con qué frecuencia ocurre lo siguiente:

|  | Nunca _1_ | A Veces _2_ | Casi Siempre _3_ | Siempre _4_ |
| --- | --- | --- | --- | --- |
| 17.¿ Cuanto comprende las recomendaciones hechas por su doctor? |  |  |  |  |
| 18. ¿Cuantas veces los medicamentos no están disponibles cuando Ud.los necesita? |  |  |  |  |
| 19. ¿Con qué frecuencia le es imposible obtener los medicamentos debido a su costo? |  |  |  |  |
| 20. ¿ Cuando se le recomienda que se haga análisis de sangre, cuántas veces puede Ud, cumplir con esa recomendación? |  |  |  |  |
| 21. ¿ Cuando se le recomienda que se haga radiografías, cuántas veces puede Ud. cumplir con esa recomendación? |  |  |  |  |
| 22.Si la persona que lo atiende lo deriva a un especialista, cuántas veces puede Ud.cumplir con esa recomendación |  |  |  |  |
